# Metabolic and Intestinal Morphometric Responses of Nile Tilapia Fed Diets Containing Soybean and Protease

**DOI:** 10.3390/ani15030349

**Published:** 2025-01-25

**Authors:** Thamara Luísa Staudt Schneider, Roberta Cristina Scheid, Nilce Coelho Peixoto, Rafael Lazzari

**Affiliations:** 1 Programa de Pós-Graduação em Zootecnia, Centro de Ciências Rurais (CCR), Universidade Federal de Santa Maria (UFSM), Campus Sede, Santa Maria 97105-900, Brazil; rlazzari@ufsm.br; 2 Curso de Graduação em Zootecnia, Universidade Federal de Santa Maria (UFSM), Campus Palmeira das Missões, Palmeira das Missões 98300-000, Brazil; scheidroberta@gmail.com; 3 Departamento de Ciências da Saúde, Universidade Federal de Santa Maria (UFSM), Campus Palmeira das Missões, Palmeira das Missões 98300-000, Brazil; ncpeixoto@yahoo.com.br; 4 Departamento de Zootecnia, Centro de Ciências Rurais (CCR), Universidade Federal de Santa Maria (UFSM), Campus Sede, Santa Maria 97105-900, Brazil

**Keywords:** aquaculture, exogenous protease, protein metabolism, *Oreochromis niloticus*

## Abstract

Proteases facilitate the breakdown of components associated with antinutritional factors. This study evaluated the effects of protease supplementation in Nile tilapia diets on metabolic and intestinal responses. Three diets were tested: soybean meal SM1 (fish meal FM:SM = 1:1), SM2 (1:2), and SM3 (1:3), based on the protein content. All the diets were without or with protease (0.44 g/kg). The fish were randomly allocated to 18 tanks/49 days. In SM3 without protease, an increase in the villus height and width, as well as the number of goblet cells, was observed. Protease supplementation increased the villus height compared to diets without the enzyme. The SM1 with protease resulted in a reduction in the digestive somatic index, but significantly increased the hepatosomatic and intestinal quotient indices. In SM2 with protease, reductions in the amino acid (AA) content and aspartate aminotransferase activity in the liver were observed. In the muscles, supplementation increased the AA concentrations. Higher plasma AA and albumin concentrations were detected in the SM2 and SM3 groups with protease. A significant interaction was found, with SM3 without protease showing lower plasma protein and AA concentrations. In SM2 with protease, higher levels of albumin and globulin were recorded. Protease in soybean meal diets improved the intestinal health and metabolism in tilapia.

## Simple Summary

Exogenous proteases play a crucial role in mitigating the effects of antinutrients from plant-based ingredients in diets. This study assessed the impact of protease supplementation in diets containing soybean meal. Tilapia fed diets with a higher plant protein inclusion and 0.44 g/kg of protease exhibited improved intestinal health and metabolic indices. Protease supplementation reduced the negative effects of plant ingredients, particularly soybean meal, providing a cost-effective alternative in diets with lower-cost ingredients.

## 1. Introduction

The increasing need to replace animal-based ingredients with plant-based alternatives has gained prominence in aquaculture, driven by a high demand, rising costs, and sustainability challenges associated with animal-derived products [1]. Plant proteins, such as those from soy and wheat, are widely available, commercially accepted, and considered promising alternatives. However, these sources often contain high levels of dietary fiber, composed of non-starch polysaccharides and lignin, which reduce digestibility [2].

In this context, diets containing plant-based ingredients, such as soybean meal, are emerging as sustainable alternatives to replace animal-derived components. While soybean meal is widely available and has a favorable amino acid profile, it also contains antinutritional factors, such as trypsin inhibitors and phytic acid, which can negatively impact protein digestibility and intestinal health [3,4]. Adverse environmental and nutritional conditions can lead to metabolic disturbances and digestive adaptations [5,6], including a reduced feed intake, impaired nutrient digestion, and an increased susceptibility to infections.

Exogenous protease supplementation has shown promise in mitigating the negative effects of antinutritional factors, enhancing digestibility, gut health, and overall fish performance [7,8]. Studies have demonstrated that the addition of proteases to soybean-meal-based diets improves protein efficiency, endogenous digestive enzyme activity, and nutrient retention, while also positively impacting the water quality in production systems [9,10,11,12]. Furthermore, the inclusion of protease reduces diet costs and enhances the economic viability of fish production [7,12].

Protease supplementation in fish diets has shown significant benefits for performance, health, and metabolism. Inclusion levels of up to 0.60 g/kg improved the growth and body composition of rohu carp (*Labeo rohita*) when poultry by-products were used in the diet [10]. In European sea bass (*Dicentrarchus labrax*), diets containing dried distillers’ grains and exogenous protease reduced the aminotransferase activity levels and promoted growth [13]. These findings underscore the potential of protease to enhance the growth and health in aquaculture species.

The Nile tilapia (*Oreochromis niloticus*) is one of the most consumed fish species worldwide, accounting for 9% of global production and ranking as the third most produced species in 2020 [14]. In Brazil, tilapia represented 63.93% of the country’s farmed fish production in 2022, marking a 3% increase compared to 2021 [15]. Its rapid growth, hardiness, and omnivorous feeding habits make it highly favorable for aquaculture systems [16,17].

This study aimed to evaluate the effects of diets containing vegetable protein and exogenous protease on the intestinal health and metabolic responses of Nile tilapia.

## 2. Materials and Methods

### 2.1. Experimental Diets

A total of six isonitrogenous [36% crude protein (CP)] and isocaloric (18 MJ/kg gross energy) experimental diets were formulated to meet the nutritional requirements of Nile tilapia (*O. niloticus*). The first diet, soybean meal (SM1), included animal-based protein sources [feather meal, poultry by-product meal, and predominantly fish waste meal (FM)] and plant-based protein sources (corn, wheat bran, and predominantly soybean meal), with an FM:SM ratio of 1:1. The second diet, SM2, had an FM:SM ratio of 1:2, and the third group, SM3, had an FM:SM ratio of 1:3, with ingredient substitutions based on the protein content. Protease was included in the diets at 0 or 0.44 g/kg. This enzyme is a serine protease (EC 3.4.21) and contains 75,000 PROT units/g (supplied by DSM Nutritional Products Ltd., Mszczonow, Maz., Poland). One PROT unit is defined as the amount of enzyme that releases 1 µmol of *p*-nitroaniline from 1 µM of substrate (Suc-Ala-Ala-Pro-Phe *p*-nitroaniline) per minute at a pH of 9.0 and a temperature of 37 °C.

The ground ingredients were mixed with oils and distilled water. The mixture was extruded in a single-screw extruder (Inbramaq, model Labor PQ 30, São Paulo, SP, Brazil). The pellets (2.0 mm diameter) were then dried in an oven with forced air recirculation at 55 °C for 24 h. Subsequently, the enzyme was added by spraying with a manual pump, and the diets were stored at −20 °C throughout the experimental period. The composition of the diets and an analysis of the enzyme recovery are presented in Table 1.

The chemical composition of the experimental diets was determined by following the methods of the Association of Official Analytical Chemists [19]. Dry matter was determined by oven drying at 105 °C until a constant weight was reached (method 934.01). The mineral matter content was estimated after the incineration of the samples in a muffle furnace at 550 °C for 4 h (method 968.08). The crude protein content (N × 6.25) was determined by the Kjeldahl method after acid digestion (method 954.01). The crude lipid content was determined by the chloroform and methanol extraction method of Bligh and Dyer [20]. The neutral detergent fiber content of the experimental diets was determined by the method described by Van Soest [21]. Nitrogen-free extracts (NFE) were calculated according to Bureau et al. [22].

### 2.2. Experimental Conditions and Fish-Feeding Management

The experiment was conducted at the Fish Farming Laboratory of UFSM, Campus Palmeira das Missões, RS, Brazil. Nile tilapia were obtained from AquaViva Commercial Fish Farm, Victor Graeff, RS, Brazil. The fish were acclimated to the experimental conditions for two weeks and fed a commercial diet (Supra™, Esteio, RS, Brazil, 36% CP). After acclimation, 360 male juvenile fish (average initial weight: 11.60 ± 0.32 g) were randomly distributed into 18 tanks (usable volume: 220 L), with a stocking density of 20 fish per tank (1 g/L). Each of the six treatments had three replicate tanks. For 49 days, their feed intake was measured daily, and the fish were manually fed until satiety, three times a day (08:00 a.m., 1:30 p.m., and 6:00 p.m.). Siphoning was performed in each tank daily (10:00 a.m. and 4:00 p.m.).

The water temperature and dissolved oxygen were measured daily using YSI ProODO technology (YSI™ Inc., Yellow Springs, OH, USA). Weekly, the total alkalinity (via neutralization titration), total hardness (via complexation titration), pH (YSI™ pH100, Yellow Springs, OH, USA), non-ionized ammonia, and nitrite levels (Alfakit™ colorimetric kit, Florianópolis, SC, Brazil) were evaluated. During the experimental period, the following water quality parameters were recorded: temperature, 25.94 ± 1.00 °C; dissolved oxygen, 5.71 ± 0.57 mg/L; pH, 7.01 ± 0.45; toxic ammonia, 0.03 ± 0.02 mg NH_3_/L; nitrite, 0.46 ± 0.09 mg NO_2_^−^/L; alkalinity, 34.62 ± 10.34 mg CaCO_3_/L; and hardness, 85.64 ± 4.45 mg CaCO_3_/L. All the parameters remained within normal limits for optimal tilapia growth [3,16].

### 2.3. Sample Collection and Analysis

At the end of the experiment, blood samples were collected from nine fish per treatment by puncturing the caudal vein using syringes soaked in heparin. The collected blood was analyzed for erythrocyte parameters. A portion of the blood sample was centrifuged at 3500 rpm at 4 °C for 10 min, and the supernatant was used for a plasma analysis.

The fish were euthanized with an anesthetic overdose [23] and spinal cord section. Samples of the liver, total muscle, and anterior intestinal tract were collected from nine fish per treatment for biochemical and morphometric analyses.

#### 2.3.1. Intestinal Morphometry

The samples were fixed in a 10% formaldehyde solution for 24 h and subsequently preserved in 70% alcohol. After routine histological processing, the tissues were embedded in paraffin, and blocks were sectioned into 5 μm slices using a microtome (Thermo Scientific™ HM 355S, Walldorf, BW, Germany). Transverse sections were stained with periodic acid-Schiff (PAS) (Sigma-Aldrich™, 3951; Saint-Louis, MO, USA), following the manufacturer’s recommended procedures and adapted from Okuthe and Bhomela [24]. The histological sections were examined under an Axio Scope A1 microscope (ZEISS™, Oberkochen, BW, Germany), photographed with an Axiocam camera, and analyzed using the ImageJ software (version 1.54d, Bethesda, MD, USA). In the anterior intestine, the height, width, and number of goblet cells in the villi were assessed (54 villi per treatment, totaling 324 villi measured).

#### 2.3.2. Morphometric Indices

Calculated based on the weight and length of the digestive tract, liver, and visceral fat using the following equations: hepatosomatic index (HSI) = [(liver weight/final weight) × 100]; digestive somatic index (DSI) = [(digestive tract weight/final weight) × 100]; celomic fat index (CFI) = [(abdominal cavity fat weight/final weight) × 100]; and intestinal quotient (IQ) = (digestive tract length/total length).

#### 2.3.3. Biochemical Tissue Analysis

Tissue samples (liver and muscle) were heated in a 6 M potassium hydroxide solution at 100 °C for 20 min to analyze the total protein [25] and glycogen [26] content. Homogenization in a 20 mM potassium phosphate buffer solution, with a pH of 7.5, followed by centrifugation at 3500 rpm for 10 min, was performed to quantify the amino acids (AAs) [27] and to assess the activity of the enzymes alanine aminotransferase (ALT) and aspartate aminotransferase (AST) [28]. For the determination of the total ammonia content [29], homogenization was performed in a 10% trichloroacetic acid solution.

#### 2.3.4. Plasma Biochemical Analysis

In plasma, the concentrations of total proteins, albumin, and glucose were analyzed using commercial colorimetric kits (Labtest™, Lagoa Santa, Brazil). Serum globulin was calculated by subtracting the albumin values from the total protein. The quantification of amino acids (AAs) was achieved using the methodology described by Spies [27].

#### 2.3.5. Erythrocyte Parameters

Blood parameters, including the erythrocyte count (RBC), hematocrit (Hct), and hemoglobin (Hb) levels, were measured [30]. The RBC count was performed after the dilution of 10 µL of blood in a formaldehyde citrate solution, and the count was performed in a Neubauer chamber (Loptik Labor™, Stuttgart, BW, Germany) with the aid of an optical microscope (Bioval™, São Paulo, SP, Brazil). Hct was determined by the microhematocrit technique. The microcapillary was filled with approximately 10 µL (5 cm) of blood and centrifuged at 12,000 rpm for 15 min, after which the cell column was measured using an Hct ruler (Benfer™, Piracicaba, SP, Brazil). The Hb was determined using a commercial colorimetric kit (Labtest™, Lagoa Santa, MG, Brazil), which involved homogenizing 20 µL of blood in a color reagent containing potassium cyanide; subsequently, the absorbance at 540 nm was determined using a spectrophotometer (Bioespectro™, Curitiba, PR, Brazil).

Erythrocyte indices, including the mean corpuscular volume (MCV), mean corpuscular hemoglobin (MCH), and mean corpuscular hemoglobin concentration (MCHC), were calculated using the following formulas: MCV = (Hct × 10)/RBC; MCH = (Hb × 10)/RBC; and MCHC = (Hb × 100)/Hct [28].

### 2.4. Statistical Analysis

All the data were analyzed using the R™ software, version 4.3.0 (R Foundation for Statistical Computing, Vienna, Austria), and graphs were created using the SigmaPlot™ software, version 14.5 (Systat Software Inc., San Jose, CA, USA). The data were subjected to a Shapiro–Wilk normality analysis. Two-way ANOVA was used to analyze the individual effects of diets and exogenous protease and the interaction among them. The Tukey test was applied if there was a significant difference (*p* < 0.05), and the results are presented as the means and standard error (±SE).

## 3. Results

### 3.1. Intestinal Morphometry

**Figure 1:**
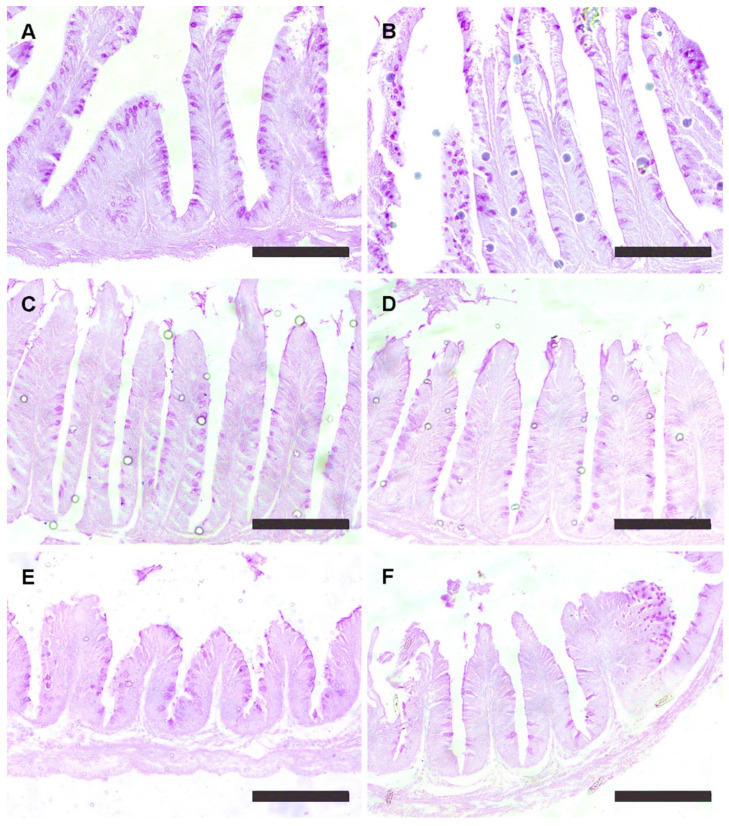
Intestinal villi of Nile tilapia fed experimental diets for 49 days. Groups: SM1 = FM:SM of 1:1; SM2 = FM:SM of 1:2; and SM3 = FM:SM of 1:3. (**A**) SM1 without protease; (**B**) SM1 with protease (0.44 g/kg); (**C**) SM2 without protease; (**D**) SM2 with protease (0.44 g/kg); (**E**) SM3 without protease; and (**F**) SM3 with protease (0.44 g/kg). SM: soybean meal, FM: fish waste meal. The sections were stained with periodic acid-Schiff. Scale bars = 300 μm.

The SM1 and SM2 groups without protease supplementation exhibited a reduced villus height, a reduced width, and a lower number of goblet cells in the anterior portion of the intestine compared to the SM3 group (Table 2). Protease supplementation resulted in greater villus height compared to groups lacking exogenous protease (Figure 1B,D,F). The SM1 group with protease showed a higher villus height than the SM3 group without protease. Conversely, the SM1 group had a lower villus width compared to the SM3 group without protease.

### 3.2. Morphometric Indices

Exogenous protease supplementation resulted in a significantly higher digestive somatic index (DSI) in fish fed the SM3 diet compared with the SM1 diet, but the DSI of these diets did not differ from that of the SM2 diet (Table 3). A significant interaction was observed in the DSI, where the SM3 group presented a higher DSI compared to the SM1 diet with protease. Protease supplementation significantly increased the hepatosomatic index (HSI) compared to diets without protease. In the SM3 group, fish fed the diet containing protease had a higher intestinal quotient (IQ) compared to the diet lacking protease. There was no significant effect of diets, exogenous protease, or interactions in the CFI.

### 3.3. Biochemical Tissue Analysis

**Figure 2:**
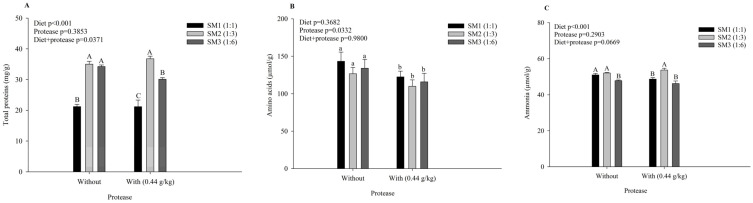

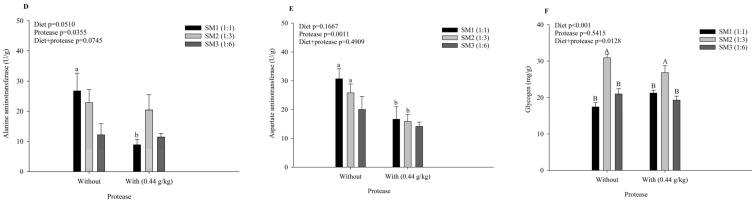
Biochemical parameters of Nile tilapia liver fed experimental diets for 49 days. Groups: SM1 = FM:SM of 1:1; SM2 = FM:SM of 1:2; and SM3 = FM:SM of 1:3, without and with protease (0.44 g/kg). (**A**) Total proteins (mg/g); (**B**) Amino acids (µmol/g); (**C**) Ammonia (µmol/g); (**D**) Alanine aminotransferase (U/g); (**E**) Aspartate aminotransferase (U/g); (**F**) Glycogen (mg/g). SM: soybean meal, FM: fish waste meal. Capital letters indicate differences between diets with the same protease supplementation, and lowercase letters indicate differences without and with protease within the same diet, according to Tukey’s test (*p* < 0.05), *n* = 6.

In the liver, the diets influenced the concentration of total proteins, ammonia, and glycogen, particularly in the SM2 group, which generally showed higher concentrations of these elements compared to the other groups (Figure 2). The supplementation of protease affected the concentration of AAs and the activity of the ALT and AST enzymes, resulting in lower concentrations of AAs and enzymatic activity compared to the diets without protease.

There was a significant interaction for the concentration of total proteins in the muscle tissue (Table 4). The SM1 group had a lower concentration of AAs and a higher ammonia content compared to the SM3 group. With protease supplementation, the concentration of AAs was higher in the SM1 and SM2 groups compared to the SM3 group. There was a significant effect of enzyme supplementation and a significant interaction for the glycogen concentration. A higher concentration was observed in the SM1 with protease and SM2 without protease groups compared to the other groups.

### 3.4. Plasma Biochemical Analysis

In general, the SM3 group had higher concentrations of AAs, total proteins, and globulin compared to the SM1 group (Table 5). The SM2 group with protease supplementation showed a higher concentration of albumin and globulin compared to the SM1 group with protease, but did not differ from the SM3 group with the enzyme.

### 3.5. Erythrocyte Parameters

There were no differences between diets, protease, or significant interactions for the hematological parameters (RBC, Hct, and Hb) or calculated blood indices (MCV, MCH, and MCHC) (Table 6).

## 4. Discussion

### 4.1. Intestinal Morphometry

In this study, reduced villus development and a lower number of goblet cells were observed in the SM1 and SM2 groups without protease. In the protease-supplemented groups (SM3), improvements in nutrient absorption parameters were observed, suggesting a potential positive effect, although digestibility was not directly proven. Two possible explanations for the reduced villus development are proposed: (a) a lower quality of ingredients due to the presence of indigestible materials in animal by-products, such as minerals, chitin, and keratinized proteins [10,31,32], and (b) the characteristics of the protease enzyme, as its action can be inhibited by a greater presence of protein from animal by-products [7,33,34,35]. The interaction between protease supplementation and animal by-products still requires further study, as well as a digestibility assessment, to better understand their synergistic effects.

The inclusion of soybean meal and the presence of antinutritional factors can cause enteritis and intestinal damage [3,4,36,37]. In this study, diets with higher levels of plant protein (SM) supplemented with protease increased the villus height and reduced the villus width, optimizing nutrient absorption. The number of goblet cells remained elevated, possibly maintaining their protective function in the fish’s intestine. Additionally, supplementation with exogenous protease reduced intestinal inflammation and improved the intestinal morphometry, thus promoting villus development and enhancing the capacity for digestion and nutrient absorption [13,38,39,40,41].

### 4.2. Morphometric Indices

The SM3 group presented a higher DSI compared to the SM1 group with protease, indicating digestive tract adaptation to improve nutrient digestibility. Enzyme supplementation influenced the liver health and intestinal status, with higher HSI values observed in diets containing 0.44 g/kg of protease. Additionally, the SM3 group with protease presented a higher IQ, suggesting increased intestinal development. The enzymatic hydrolysis of proteins may lead to both liver overload and enhanced intestinal adaptation [12,39,40]. Protease supplementation has demonstrated clear benefits for nutrient digestibility in non-ruminant diets [8,9], and its combination with other enzymes has been shown to enhance the digestive tract function in Nile tilapia [41,42]. These findings collectively indicate that protease positively influences metabolic responses and intestinal morphometry, particularly in diets with higher levels of plant protein (SM3 group).

### 4.3. Biochemical Parameters in Tissues

The diets in this study significantly affected the levels of total proteins, ammonia, and glycogen in Nile tilapia, with a significant interaction observed in the total protein and glycogen levels. Increased levels of proteins and AAs are associated with protein metabolism and can negatively influence growth if the diet is imbalanced [43]. The SM2 group exhibited higher concentrations of total proteins and glycogen in the liver, with no statistical difference from the SM1 group. This suggests that the greater inclusion of vegetable protein may favor glycogen and protein reserve formation. Previous studies have indicated that diets deficient in essential AAs can impair liver function, reducing aminotransferase enzyme activity [3,44]. However, no negative effects on liver health were observed in this study, as the transaminase enzyme activity remained unaffected by the diets.

Previous studies have shown that exogenous protease improves AA utilization in various carp species, reducing blood ammonia concentrations and increasing the aminotransferase enzyme activity in the liver [39]. This suggests that exogenous protease increases AA availability, promoting the synthesis of small peptides and AAs through aminotransferase activity [3,45]. The lack of essential AAs and peptides in the diet has been shown to compromise protein metabolism, leading to decreased growth in turbot [43] and failures in protein synthesis in silvery-black porgy (*Sparidentex hasta*) [44]. In the present study, fish fed diets containing protease showed lower AA concentrations and aminotransferase enzyme activity, suggesting that protease improves the protein metabolism efficiency. Additionally, diets with higher plant protein inclusion (SM3), even with protease, did not negatively affect the liver health, as indicated by lower aminotransferase concentrations. This suggests that protease, combined with a balanced AA supply, provides hepatic metabolic balance in diets containing vegetable protein.

In this study, the total concentration of proteins in the muscle was influenced exclusively by their interaction. Notably, the SM1 group exhibited a higher concentration of total proteins compared to the SM2 and SM3 groups, particularly when the enzyme was not included. The effect of protease on diets that meet the nutritional requirements of the species and have a high digestibility was not observed in studies with gibel carp (*Carassius auratus*) [34] or Pacific white shrimp (*Litopenaeus vannamei*) [35]. In this study, the effect of diets and their interaction on the AA and ammonia concentrations was observed. In the absence of exogenous protease, the SM2 group exhibited higher AA and glycogen concentrations and a lower ammonia content compared to the SM1 group. The significant interaction between diet composition and enzyme supplementation suggests that balancing protein sources, particularly with protease inclusion, can optimize nutrient utilization and metabolic processes in fish muscle. Furthermore, these results, along with the absence of aminotransferase enzyme activity in the muscle and the lower AA concentration in the liver, possibly indicate a better energy reserve balance in the FM:SM ratio of 1:3 (SM3).

### 4.4. Plasma Biochemical Parameters

In the present study, the plasma glucose levels were not affected by the experimental conditions, similar to Nile tilapia [12]. The SM3 group presented a higher concentration of total proteins and AAs than the SM1 group, with no differences concerning the SM2 group. This suggests an increase in anabolic processes due to the greater availability of total proteins and AAs [42,46]. Plasma albumin, which is responsible for the storage and transport of AAs, correlates with their availability [47]. Previous studies have shown that elevated albumin levels are associated with stress conditions and immunostimulatory responses in different diets [13,42,46]. In the present study, the SM2 group with protease supplementation exhibited higher concentrations of albumin and globulin compared to the SM1 group, with no significant differences relative to the SM3 group. The SM3 group, which included more plant protein and was supplemented with protease, demonstrated an increase in the availability of AAs, favoring the synthesis of plasma proteins, such as albumin and globulin. These results suggest a metabolic and immunological adaptation of fish to diets with higher proportions of plant protein.

### 4.5. Erythrocyte Parameters

Blood indices are important for monitoring fish health. Previous studies have shown that the high supplementation of plant protein can result in anemia indicators [6]. In this study, no influence of diets or exogenous protease was observed on the erythrocyte parameters. Previous research on Nile tilapia and striped catfish (*Pangasius hypophthalmus*) also did not find significant differences related to protease supplementation in diets [12,31]. Diets with lower-quality ingredients containing protease did not negatively affect fish health [34,42,46]. The blood parameters remained within the reference ranges for Nile tilapia, indicating good fish health under the experimental conditions [48].

## 5. Conclusions

In conclusion, protease supplementation at 0.44 g/kg in diets with higher soybean meal inclusion improved the intestinal health and nutrient absorption efficiency of Nile tilapia.

## Figures and Tables

**Table 1:** Composition of the diets of Nile tilapia.

Ingredient (%)	Diet					
	SM1 (1:1)		SM2 (1:2)		SM3 (1:3)	
	SM1	SM1 + Protease	SM2	SM2 + Protease	SM3	SM3 + Protease
Fish waste meal (FM)	15	15	10	10	5	5
Feather meal	8.5	8.5	8.5	8.5	8.5	8.5
Poultry by-product meal	4	4	4	4	4	4
Soybean meal (SM)	32	32	40	40	47	47
Corn	28	28	28	28	28	28
Wheat bran	7.23	7.23	4.23	4.23	2.23	2.23
Soybean and canola oil (1:1)	2	2	2	2	2	2
Vitamins and minerals ^a^	1.5	1.5	1.5	1.5	1.5	1.5
Ascorbic acid	0.05	0.05	0.05	0.05	0.05	0.05
Salt	0.5	0.5	0.5	0.5	0.5	0.5
Methionine	0.2	0.2	0.2	0.2	0.2	0.2
Starch	1	1	1	1	1	1
Antioxidant ^b^	0.02	0.02	0.02	0.02	0.02	0.02
Analyzed composition (%)						
Dry matter	94.24 ± 0.13	92.03 ± 1.38	93.52 ± 0.09	94.02 ± 0.25	93.27 ± 0.14	93.84 ± 0.24
Mineral matter	8.62 ± 0.06	7.75 ± 0.52	7.43 ± 0.40	7.33 ± 0.34	6.85 ± 0.07	6.67 ± 0.11
Crude protein	36.69 ± 0.61	35.21 ± 0.73	35.87 ± 0.31	36.73 ± 0.07	35.79 ± 0.44	35.38 ± 0.49
Lysine ^c^	1.97	1.97	1.98	1.98	1.98	1.98
Methionine ^c^	0.74	0.74	0.70	0.70	0.66	0.66
Crude lipid	7.34 ± 0.34	6.71 ± 0.16	7.12 ± 0.19	7.56 ± 0.34	6.76 ± 0.25	6.54 ± 0.33
Neutral detergent fiber	14.33 ± 0.92	14.33 ± 0.92	10.42 ± 1.74	10.42 ± 1.74	11.08 ± 0.94	11.08 ± 0.94
NFE ^d^	33.02	36.00	39.16	37.96	39.52	40.33
Trypsin inhibitor (mg) ^e^	1.28	1.28	1.60	1.60	1.88	1.88
Phytic acid (mg) ^e^	4.80	4.80	6.00	6.00	7.05	7.05
Gross energy (MJ/kg) ^f^	17.23	17.15	18.01	18.18	17.91	17.86
Protease (prot/kg) ^g^	0	27,390	0	25,930	0	21,370

^a^ Composition (kg/product): folic acid, 370 mg; pantothenic acid, 3900 mg; biotin, 40 mg; cobalt, 58 mg; copper, 740 mg; choline, 75 g; iron, 7500 mg; inositol, 10 g; iodine, 43 mg; manganese, 7800 mg; niacin, 8800 mg; selenium, 38 mg; vitamin A, 780,000 IU; vitamin B1, 1400 mg; vitamin B12, 1900 mcg; vitamin B2, 1450 mg; vitamin B6, 1400 mg; vitamin C, 19.5 g; vitamin D3, 160,000 IU; vitamin E, 14,800 IU; vitamin K3, 475 mg; zinc, 1400 mg. ^b^ Butylated hydroxytoluene. ^c^ Calculated based on the aminogram of the ingredients. ^d^ Nitrogen-free extracts = [100 − (crude protein + lipids + mineral matter + crude content)]. ^e^ Calculated based on soybean meal as described by Francis et al. [2]. ^f^ Gross energy calculated using gross calorific values of 23.63, 39.52, and 17.15 kJ/g for protein, fat, and carbohydrates [18]. ^g^ Relative activity of protease analyzed by BIOPRACT^™^ GmbH (Berlin, Germany).

**Table 2:** Intestinal morphometry and number of goblet cells of Nile tilapia fed experimental diets for 49 days.

Group	Protease (g/kg)	Intestine		
		Villus Height (µm)	Villus Width (µm)	Goblet Cells (Units/Villus)
SM1 (1:1)	0	185.35 ± 3.70 ^Cb^	79.18 ± 2.21 ^B^	83 ± 4.47 ^B^
	0.44	274.25 ± 5.58 ^Ba^	77.54 ± 3.00 ^B^	78 ± 5.33
SM2 (1:2)	0	210.46 ± 5.24 ^Bb^	77.27 ± 2.43 ^Bb^	76 ± 8.50 ^B^
	0.44	269.76 ± 7.10 ^Ba^	87.02 ± 3.30 ^Aa^	90 ± 3.14
SM3 (1:3)	0	254.54 ± 4.29 ^Ab^	91.15 ± 3.58 ^Aa^	102 ± 2.59 ^A^
	0.44	295.81 ± 3.71 ^Aa^	80.24 ± 2.19 ^ABb^	95 ± 7.01
Two-way ANOVA				
Diet		<0.001	0.0369	0.0020
Protease		<0.001	0.6867	0.9115
Diet + protease		<0.001	0.0015	0.1010

Capital letters indicate differences between diets with the same protease supplementation, and lowercase letters indicate differences without and with protease within the same diet, according to Tukey’s test (*p* < 0.05), *n* = 7. Group SM1: FM:SM of 1:1 without and with protease (0.44 g/kg); SM2 group: FM:SM of 1:2 without and with protease (0.44 g/kg); SM3 group: FM:SM of 1:3 without and with protease (0.44 g/kg). SM: soybean meal, FM: fish waste meal.

**Table 3:** Morphometric indices of Nile tilapia fed experimental diets for 49 days.

Group	Protease (g/kg)	HSI (%)	DSI (%)	CFI (%)	IQ
SM1 (1:1)	0	1.84 ± 0.10 ^b^	6.56 ± 0.46	1.89 ± 0.12	6.04 ± 0.28
	0.44	2.19 ± 0.07 ^a^	5.49 ± 0.40 ^B^	1.78 ± 0.30	6.32 ± 0.17
SM2 (1:2)	0	2.00 ± 0.04 ^b^	5.61 ± 0.32	1.72 ± 0.31	6.24 ± 0.14
	0.44	2.25 ± 0.08 ^a^	6.66 ± 0.34 ^AB^	1.35 ± 0.20	6.36 ± 0.11
SM3 (1:3)	0	1.72 ± 0.12 ^b^	6.69 ± 0.22	1.61 ± 0.18	6.15 ± 0.20
	0.44	2.12 ± 0.06 ^a^	7.58 ± 0.28 ^A^	1.24 ± 0.11	7.11 ± 0.20
Two-way ANOVA					
Diet		0.0600	0.0055	0.1697	0.0717
Protease		<0.001	0.3181	0.1168	0.0079
Diet + protease		0.6016	0.0075	0.7959	0.0877

Capital letters indicate differences between diets with the same protease supplementation, and lowercase letters indicate differences without and with protease within the same diet, according to Tukey’s test (*p* < 0.05), *n* = 6. Group SM1: FM:SM of 1:1 without and with protease (0.44 g/kg). Group SM2: FM:SM of 1:2 without and with protease (0.44 g/kg). Group SM3: FM:SM of 1:3 without and with protease (0.44 g/kg). SM: soybean meal, FM: fish waste meal. HSI: hepatosomatic index; DSI: digestive somatic index; CFI: celomic fat index; IQ: intestinal quotient.

**Table 4:** Biochemical parameters in the muscle of Nile tilapia fed experimental diets for 49 days.

Group	Protease (g/kg)	Total Proteins (mg/g)	Amino Acids (µmol/g)	Ammonia (µmol/g)	ALT (U/g)	AST (U/g)	Glycogen (mg/g)
SM1 (1:1)	0	29.49 ± 1.21	37.86 ± 3.21 ^Cb^	13.79 ± 0.47 ^A^	5.06 ± 0.55	87.64 ± 8.16	24.15 ± 0.45 ^b^
	0.44	36.46 ± 1.58	53.70 ± 0.84 ^Ba^	12.64 ± 0.26 ^A^	6.15 ± 0.65	84.60 ± 11.53	26.12 ± 0.52 ^a^
SM2 (1:2)	0	36.93 ± 0.79	52.12 ± 2.30 ^Bb^	11.57 ± 0.11 ^B^	5.12 ± 0.70	74.58 ± 5.95	26.81 ± 0.39 ^a^
	0.44	27.09 ± 1.22	78.21 ± 3.22 ^Aa^	12.94 ± 0.28 ^A^	3.51 ± 0.37	73.54 ± 4.25	21.07 ± 0.79 ^b^
SM3 (1:3)	0	30.79 ± 0.71	73.61 ± 5.99 ^Aa^	11.47 ± 0.11 ^B^	4.17 ± 0.81	98.05 ± 17.84	25.22 ± 0.62
	0.44	29.96 ± 1.00	62.74 ± 2.48 ^Bb^	11.57 ± 0.10 ^B^	5.03 ± 0.85	75.03 ± 5.79	24.66 ± 0.47
Two-way ANOVA							
Diet		0.0813	<0.001	<0.001	0.1501	0.3810	0.0868
Protease		0.1898	<0.001	0.6027	0.8421	0.2792	0.0034
Diet + protease		<0.001	<0.001	<0.001	0.1021	0.4890	<0.001

Capital letters indicate differences between diets with the same protease supplementation, and lowercase letters indicate differences without and with protease within the same diet, according to Tukey’s test (*p* < 0.05), *n* = 6. SM1 group: FM:SM of 1:1 without and with protease (0.44 g/kg); SM2 group: FM:SM of 1:2 without and with protease (0.44 g/kg); SM3 group: FM:SM of 1:3 without and with protease (0.44 g/kg). SM: soybean meal, FM: fish waste meal. ALT: alanine aminotransferase; AST: aspartate aminotransferase.

**Table 5:** Biochemical parameters in the plasma of Nile tilapia fed experimental diets for 49 days.

Group	Protease (g/kg)	Glucose (mg/dL)	Total Proteins (g/dL)	Amino Acids (µmol/dL)	Albumin (g/dL)	Globulin (g/dL)
SM1 (1:1)	0	59.97 ± 7.43	3.55 ± 0.07 ^B^	1944.66 ± 30.39 ^B^	1.18 ± 0.03	2.28 ± 0.04 ^B^
	0.44	50.85 ± 3.95	3.41 ± 0.06 ^B^	1908.65 ± 94.01 ^B^	1.05 ± 0.07 ^B^	2.27 ± 0.04 ^B^
SM2 (1:2)	0	59.24 ± 5.62	3.70 ± 0.12 ^AB^	2027.64 ± 294.46 ^B^	1.32 ± 0.12	2.46 ± 0.08 ^AB^
	0.44	54.62 ± 4.71	3.90 ± 0.06 ^A^	2407.34 ± 82.86 ^AB^	1.36 ± 0.08 ^A^	2.63 ± 0.06 ^A^
SM3 (1:3)	0	49.92 ± 4.46	4.06 ± 0.12 ^A^	2819.65 ± 101.84 ^A^	1.18 ± 0.03	2.71 ± 0.08 ^A^
	0.44	53.24 ± 1.91	4.02 ± 0.19 ^A^	2454.31 ± 93.08 ^A^	1.19 ± 0.03 ^AB^	2.51 ± 0.10 ^AB^
Two-way ANOVA						
Diet		0.5515	<0.001	<0.001	0.0168	<0.001
Protease		0.4037	0.9433	0.9517	0.6397	0.7962
Diet + protease		0.4638	0.3144	0.0572	0.4064	0.0525

Capital letters indicate differences between diets with the same protease supplementation, according to Tukey’s test (*p* < 0.05), *n* = 4. SM1 group: FM:SM of 1:1 without and with protease (0.44 g/kg); SM2 group: FM:SM of 1:2 without and with protease (0.44 g/kg); and SM3 group: FM:SM of 1:3 without and with protease (0.44 g/kg). SM: soybean meal, FM: fish waste meal.

**Table 6:** Erythrocyte parameters of Nile tilapia fed experimental diets for 49 days.

Group	Protease (g/kg)	RBC (×10^6^/µL)	Hct (%)	Hb (g/dL)	MCV (fL)	MCH (pg)	MCHC (g/dL)
SM1 (1:1)	0	1.16 ± 0.10	34.30 ± 1.00	7.69 ± 0.31	306.96 ± 25.91	66.42 ± 6.08	22.47 ± 1.05
	0.44	0.97 ± 0.08	35.95 ± 1.13	7.84 ± 0.32	385.20 ± 27.99	77.10 ± 5.69	21.59 ± 1.19
SM2 (1:2)	0	1.14 ± 0.10	37.30 ± 1.00	8.08 ± 0.36	350.09 ± 25.91	78.72 ± 6.57	21.92 ± 1.19
	0.44	1.03 ± 0.08	34.74 ± 1.00	8.02 ± 0.32	338.41 ± 25.91	81.17 ± 5.69	22.28 ± 1.05
SM3 (1:3)	0	1.02 ± 0.10	34.83 ± 1.00	7.19 ± 0.36	338.78 ± 25.91	76.20 ± 6.57	21.93 ± 1.12
	0.44	1.10 ± 0.11	33.96 ± 1.00	7.48 ± 0.34	292.09 ± 34.28	56.92 ± 8.05	21.23 ± 1.05
Two-way ANOVA							
Diet		0.9597	0.2764	0.1267	0.5016	0.1137	0.8549
Protease		0.3357	0.4829	0.6446	0.7680	0.6892	0.6298
Diet + protease		0.3998	0.1353	0.8721	0.0873	0.0792	0.8120

SM1 group: FM:SM of 1:1 without and with protease (0.44 g/kg); SM2 group: FM:SM of 1:2 without and with protease (0.44 g/kg); SM3 group: FM:SM of 1:3 without and with protease (0.44 g/kg). SM: soybean meal, FM: fish waste meal. *n* = 6. RBC: red blood cells; Hct: hematocrit; Hb: hemoglobin; MCV: mean corpuscular volume; MCH: mean corpuscular hemoglobin; MCHC: mean corpuscular hemoglobin concentration.

## Data Availability

The original contributions presented in the study are included in the article, further inquiries can be directed to the corresponding author.
